# Genetic diversity of *Plasmodium falciparum* helical interspersed subtelomeric* (phistb) gene* in Tanzania and neighboring countries

**DOI:** 10.1186/s12936-026-06011-x

**Published:** 2026-06-24

**Authors:** Ruth B. Mbwambo, Dativa Pereus, Catherine Bakari, Ramadhan Moshi, Angelina J. Kisambale, Rashid A. Madebe, Seth D. Misago, Celine I. Mandara, Beatus Lyimo, Daniel Mbwambo, Sijenunu Aaron, Abdallah Lusasi, Samwel Lazaro, Gerald Juma, Benard W. Kulohoma, Deus S. Ishengoma

**Affiliations:** 1 Muhimbili University of Health and Allied Sciences, Dar es Salaam, Tanzania; 2 National Institute for Medical Research, Dar es Salaam, Tanzania; 3 University of Nairobi, Nairobi, Kenya; 4 Swiss Tropical and Public Health Institute, Basel, Switzerland; 5 Nelson Mandela African Institution of Science and Technology, Arusha, Tanzania; 6 National Malaria Control Program, Dodoma, Tanzania; 7 Ortholog, Nairobi, Kenya; 8 Ifakara Health Institute, Dar es Salaam, Tanzania

**Keywords:** Malaria vaccine, Genetic diversity, *Phistb* gene, *Plasmodium falciparum*, Tanzania

## Abstract

**Background:**

Lysine-rich membrane associated *Plasmodium* helical interspersed subtelomeric gene (*phistb*) is a member of the *phist* family of genes which encodes exported proteins essential for the parasite’s survival within infected red blood cells. Recent studies suggest the *phistb* gene as a promising malaria vaccine candidate, however, its genetic diversity remains understudied. This study assessed the genetic diversity of the *phistb* gene in regions of varying malaria transmission aiming to generate data and improve our understanding of this promising malaria vaccine candidate gene.

**Methods:**

Genomic data from 1472 *Plasmodium falciparum* samples from Tanzania, Kenya, Uganda, and Ethiopia were retrieved in variant Calling file format (VCF) format from the MalariaGEN Pf7 database. Variants were filtered to include only biallelic Single Nucleotide Polymorphism (SNPs) with Variant Quality Score Log- Odds (VQSLOD) > 1 and “PASS” status. Genetic diversity, differentiation, and selection signatures were analyzed using population genetics metrics.

**Results:**

After filtering, 1312 samples were retained. Wright’s inbreeding coefficient (Fws) showed that 875 (66.7%) samples had monoclonal infections, with the highest proportion of monoclonal infections in Ethiopia (95.3%), followed by Tanzania (67.2%), Kenya (65.7%), and Uganda (50%). Among the 875 monoclonal samples, 88 haplotypes were identified, with Hap_1 (renamed PF3D7) and Hap_13 comprising 37.9 and 21.5 of the samples, respectively. Nucleotide and haplotype diversity were relatively higher in Kenya with 0.097, and 0.88 respectively, compared to the other study populations. The overall fixation index (Fst) was < 0.05, and Principal Component Analysis revealed no clear population sub-structure among countries. Negative Tajima’s D values in Tanzania, Kenya, and Ethiopia indicated an excess of low-frequency alleles.

**Conclusion:**

This study reports low genetic diversity of the *phistb* gene in the four countries despite varying malaria transmission intensities among them, thus making it a suitable candidate gene for malaria vaccine. Further studies should be conducted to assess individual antibodies recognition of the *phistb* variants and the ability to elicit cross reactivity to further support its potential as a vaccine candidate.

**Supplementary Information:**

The online version contains supplementary material available at https://doi.org/10.1186/s12936-026-06011-x.

## Background

Despite the ongoing global efforts to eliminate malaria, it remains a global health challenge particularly in Sub-Sahara Africa (SSA), where it accounted for 95% of cases and 96% of deaths in 2024. Tanzania contributed 3.3% and 4.3% of the global malaria cases and deaths, respectively [1]. Malaria cases and deaths remain relatively higher than the situation before the COVID19 pandemic, driven by both biological and non-biological challenges. The biological threats include resistance to insecticides and antimalarial drugs, diagnostics evasion due to deletions of histidine rich protein 2/3 (*hrp2/3*) genes and the emergency of the invasive vector, *Anopheles stephensi* [2–7]. The non-biological factors include climate change, health system issues, poverty, insecurity and human conflicts, reduced funding for malaria and inconsistency of political will to sustain the momentum towards malaria elimination and the global targets of eradication by 2050 [8–10].

In malaria-endemic regions, high genetic diversity of *Plasmodium falciparum* parasites arises from heterologous recombination of different parasite clones carried by infected mosquitoes, resulting from co-transmission and/or super-transmission occurring during a mosquito blood meal [11]. Genetic diversity influences parasite phenotypes including antimalarial drug resistance, virulence and poor effectiveness of malaria vaccines, thus understanding the genetic diversity of the malaria parasites is crucial for effective malaria control and elimination [12].

The RTS,S and R21 are the first malaria vaccines to be licensed by the World Health Organization (WHO), to protect children in high-risk areas. However, their efficacy is limited against parasite strains that differ from the vaccine haplotypes [13–17]. This highlights the need to assess genetic diversity of the potential malaria vaccine candidate genes for the development of broadly protective vaccines. Different studies have assessed CSP which is the target of RTS,S and R21, and other potential vaccine candidates such as reticulocyte-binding protein homologue 5 (rh5) [14, 18–20] and generate important evidence showing that some can potentially be considered for vaccine development. Thus, more studies are still needed including assessing the genetic diversity of Lysine-rich membrane-associated *Plasmodium* helical interspersed subtelomeric (*phistb*) gene [21], to generate evidence on their suitability for vaccine development.

The *phistb* gene (PF3D7_0532400) belongs to the family of *phist* genes that hosts up to 65 identified genes [21]. The PHIST protein family is classified into three groups; PHISTa, PHISTb, and PHISTc, based on the presence and the length of the amino acid tryptophan residues [22]. The PHIST proteins are part of the malaria parasites exported proteins and they play vital roles during the erythrocytic stages of the parasite's life cycle. The PHIST domain of the *phistb* gene, interacts with the intracellular acidic terminal (ATS) domain of the *P. falciparum* Erythrocyte Membrane Protein 1 (PfEMP1), helping to increase adherence and rigidity of the infected red blood cells (iRBC) [23, 24]. A recent study identified a trans-membrane domain and a Glycophosphatidylinositol (GPI) anchor suggesting that PHIST proteins are anchored on the surface of iRBCs, thus, they can be easily exposed to the human immune system [25]. In addition, the *phistb* gene is highly conserved among the main human malaria parasite species including *P. falciparum, Plasmodium vivax,* and *Plasmodium knowlesi*, thus making the gene a suitable candidate for malaria vaccine development [26]*.*

Despite that, little is known regarding the genetic diversity of the *phistb* gene, particularly in areas of different malaria endemicity and selection pressure caused by interventions such as antimalarial drugs. Previous studies that assessed the genetic diversity of the *phist* genes were limited in study sites and sample sizes. Therefore, this study expands knowledge on the genetic diversity of the *phistb* gene, in Tanzania and neighboring countries and provides useful knowledge on its potential for possible inclusion in the ongoing efforts and strategies for the development of an effective malaria vaccine.

## Material and methods

### Study sites

**Fig. 1:**
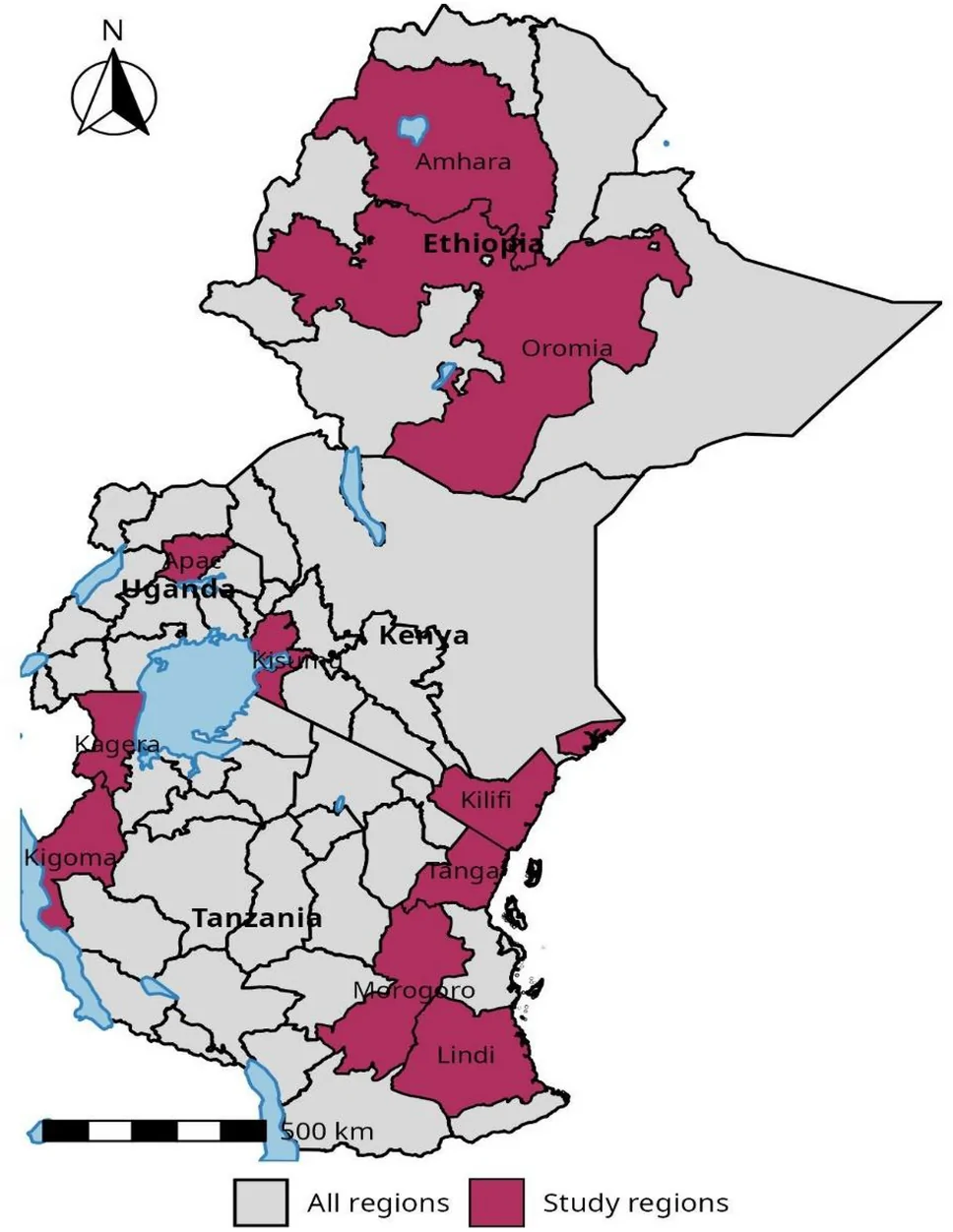
Map of Africa showing the four countries that contributed data along with the study sites in each country

This study utilized publicly available *P. falciparum* genomic data from studies that were conducted previously in Tanzania, Kenya, Uganda and Ethiopia under the Pathogen Diversity Network Africa (PDNA), as part of the Malaria Genetic Epidemiology Network’s (MalariaGEN) *P. falciparum* community project [27]. In Tanzania, the study sites included Muheza district in Tanga region, and Morogoro urban in Morogoro region, both categorized as moderate transmission settings, and Muleba district in Kagera, Nachingwea in Lindi, and Ujiji in Kigoma, which were included as high transmission regions [28]. In Kenya, the data were previously collected from Kilifi and Kisumu counties, on the coast of the Indian Ocean and near Lake Victoria, respectively [29–32]. The Apac district in Uganda [33], and Amhara and Oromia regions in Ethiopia were also among the study sites [34] as elaborated in Fig. 1.

### Study design and sampling

Genomic data used in this study was obtained from the Pf7 version of the MalariaGEN database (https://apps.malariagen.net/apps/pf7). The samples and meta-data were collected previously between 1994 and 2018, from studies conducted in Tanzania, Kenya, Uganda and Ethiopia. Details regarding the study designs, participants enrolment, inclusion and exclusion criteria for each contributing study have been reported elsewhere [28, 32–38] and summarized in the supplementary materials (Supplementary data, Table S1).

### Sample size

Genomic data in the *P. falciparum* parasite’s chromosome 5 were retrieved in Variant calling file format (VCF) from the MalariaGEN database (Pf7). The data were previously obtained from Mainland Tanzania in the five study sites including Muheza (n = 324), Muleba (n = 61), Nachingwea (n = 79), Morogoro (n = 34) and Ujiji (n = 199), while data from Kenya were from Kilifi (n = 662) and Kisumu (n = 64), Uganda from Apac (n = 15), and Ethiopia from Amhara (n = 15) and Oromia (n = 19).

### Data retrieval and quality checking

The *phistb* gene information including its chromosomal location, gene length, coding and non-coding regions were clarified from the PlasmodDB database, www.plasmodb.org [39] to ensure accuracy in the downstream analysis. Thereafter, the gene was extracted from chromosome 5 (position: 1311992–1315355) using bcf tools v1.9 [40] and filtered to retain biallelic single nucleotide polymorphisms (SNPs) that had “pass” status under the filter column of the VCF file [41], and with the variant quality score recalibration(VQSLOD) > 1. VCFtools were used to assess per-site missingness from the filtered VCF file, and a plot of per-site missingness against genomic positions was generated in R. Picard tool (v1.9) and GATK (v4.4.0) software was utilized to convert the SNPs data contained in the VCF files into FASTA sequences using the Pf3D7 *P. falciparum* reference genome. Multiple sequence alignment was done using MAFFT software (v7.305) to prepare the sequences for subsequent analysis [42].

### Within-host parasites genetic diversity analysis

The Wright’s inbreeding coefficient (Fws) was used to determine individual’s parasite genetic diversity, using the MOIMIX package [43]. All the re-constructed sequences (n = 1312) from Tanzania, Kenya, Uganda and Ethiopia were used for this analysis. The Fws metrics assess heterozygosity of parasites within an individual (HW) in comparison to the heterozygosity within a parasite population (HS), for each read count of alleles. The Fws values range from 0 to 1, whereby values close to one indicate high inbreeding and thus low genetic diversity of the parasites. In this study, Fws value ≥ 0.95 was used to indicate clonal (single strain) infection within an individual while Fws values ≤ 0.95 indicated multi-clonal (more than one parasite strain) infection [44]. The mean Fws values for each country and their study sites were plotted using the ggplot2 [45] package in R software. Moreover, the Fws values calculated from the *phistb* gene were compared with the whole-genome based Fws values generated by the MalariaGEN community. The proportion of monoclonal samples was statistically compared for both *phistb*-based and whole-genome based Fws values, using a t-test, at a 95% confidence interval. Agreement between the two methods was assessed using a 2 × 2 contingency table comparing sample-level classifications.

### Genetic diversity analysis

All of the re-constructed sequences (n = 1,312) were used for *phistb* genetic diversity analysis. A customized R script was used to read the aligned sequences into R software. Then the number of segregating sites, SNPs density and distribution along the *phistb* gene were determined using the Adegenet (v2.1.10) [43] and Ape (v5.7) [44] packages. The determined SNPs density was then plotted against their actual positions along the *phistb* gene. The DnaSP software (6.12.03) was used to assess nucleotide and haplotype diversity for each studied population and their sub-populations. To get a deeper insight of the genetic diversity in different gene loci, VCFtools were used to assess the nucleotide diversity of the *phistb* gene at a window size of 500 bp, and a step size of 50 bp then a line plot of nucleotide diversity against the corresponding midpoint of each window was generated for each studied population.

### Haplotype network construction

Monoclonal samples (875; 66.7%) were identified from all the studied populations and used for haplotype network analysis. The *phistb* gene reference sequence was retrieved from the PlasmodB database and included in the FASTA sequences containing all the monoclonal sequences (n = 875). The number of haplotypes for each studied population, and the frequency of occurrence of that particular haplotype were generated using the DnaSP software (6.12.03) [46], and used for haplotype networks construction using the median joining algorithm in the PopArt software [47].

### Population differentiation and structure analysis

The Wright’s Fixation index (Fst) was used to determine the *P. falciparum* genetic differentiation among the sub-populations (Tanga, Kagera, Lindi, Morogoro, Kigoma, Apac, Oromia, Amhara, Kilifi and Kisumu). The Nei’s pairwise Fst was estimated using the Hierfstat package in R (6.12.03), then a heatmap was generated to clearly compare the genetic differentiation across different sub-populations. Population structure analysis was done to determine clustering patterns of variants in the Tanzanian, Kenyan, Ugandan and Ethiopian parasite populations. The PLINK software (version 1.9) [48] was used to generate principal components which were then used for Principal Component Analysis (PCA) in R software.

### Signature of selection

To assess the neutrality of the *phistb* gene, Tajima’s D, Fu and Li’s F and D test were calculated for all the 1312 sequences from Tanzania, Kenya, Uganda and Ethiopia using DnaSp software version (6.12.03). Overall neutrality test values were generated for each population and tabulated. To gain a deeper understanding of the neutrality of the *phistb* gene across different loci, VCFtools software (0.1.16) was used to assess Tajima’s D in different nucleotide positions, using a window size of 1000 bp. The resulting values were then plotted for each population. Moreover, the ratio of synonymous and nonsynonymous mutations was assessed to further understand the selective pressure acting on the *phistb* gene. Briefly, Tajima’s D values were calculated separately for both synonymous and non-synonymous sites, then the ratio of the Tajima’s D for the nonsynonymous and synonymous sites was then evaluated across all the 1312 sequences, using the DnaSP software version (6.12.03) [49].

## Results

### Data filtering and quality check

Sequences used in this study were retrieved from the MalariaGEN database (Table 1) and briefly, 1472 sequences were retrieved with majority of the samples from Kenya (49.3%, 726/1,472) and Tanzania (47.3%, 697/1,472), while Uganda (1.0%, 15/1,472) and Ethiopia (2.3%, 34/1,472) contributed fewer samples. In Tanzania, most of the samples (47.3%) were from Muheza (46.4%, 324/697) and Ujiji (28.5%, 199/697), with relatively fewer samples from other sites of Muleba (8.7%, 61/697), Nachingwea (11.3%, 79/695) and Morogoro (4.9%, 34/697). In Kenya, the majority of the samples were from Kilifi (91%, 662/726), while Kisumu contributed 8.8% (64/726) of the Kenyan samples. In Ethiopia, 55.8% (19/34) and 44.1% (15/34) of the samples were from Oromia and Amhara region, respectively, while all the samples from Uganda (100%, n = 15) came from Apac district. After filtering the VCF data, 313 biallelic SNPs with the GATK’s VQSLOD > 1 were retained out of the unfiltered 832 SNPs, and used for the next analysis steps. Assessment of per-site missingness on the filtered biallelic VCF showed at least 25 sites with missing and with a data proportion greater than 0.05 (Supplementary Fig. 1). The number of samples that remained were 1,312 out of 1,472 (89.1%) where 589 (44.8%) samples were from Tanzania, 690 (52.5%) from Kenya, together with 21 (1.6%) and 12 (0.9%) from Ethiopia and Uganda, respectively.

**Table 1:** Details of samples used in this study

Country	Region	Year of collection	Before filtering, n (%)	After filtering, n (%)	Filtered total, n (%)**
Ethiopia (n = 34, 2.3%)	Amhara	2015	14 (41.2%)	10 (47.6%)	21 (1.6%)
	Oromia	2013	19 (55.9%)	11 (52.3%)	
Kenya (n = 726, 49.3%)	Kilifi	1994–2018	662 (91.2%)	627 (90.8%)	690 (52.5%)
	Kisumu	2014	64 (9.1%)	63 (9.1%)	
Tanzania (n = 697, 47.3%)	Kagera	2013	61 (8.7%)	52 (8.8%)	589 (44.8%)
	Kigoma	2014	199 (28.5%)	143 (24.2%)	
	Lindi	2013	79 (11.3%)	65 (11.0%)	
	Morogoro	2010	34 (4.8%)	32 (5.4%)	
	Tanga	2010–2014	324 (46.4%)	297 (50.4%)	
Uganda (n = 15, 1.0%)	Apac	2010	15 (100%)	12 (100%)	12 (0.9%)
Total			1472	1312	1312 (89.1%)

^**^After filtering, the number of samples were 21 (61.7%) in Ethiopia, 690 (95.0%) in Kenya, 589 (84.5%) in Tanzania and 12 (80%) in Uganda, with more loss of samples in Ethiopia and Uganda

### Within-host parasite diversity estimation

**Fig. 2:**
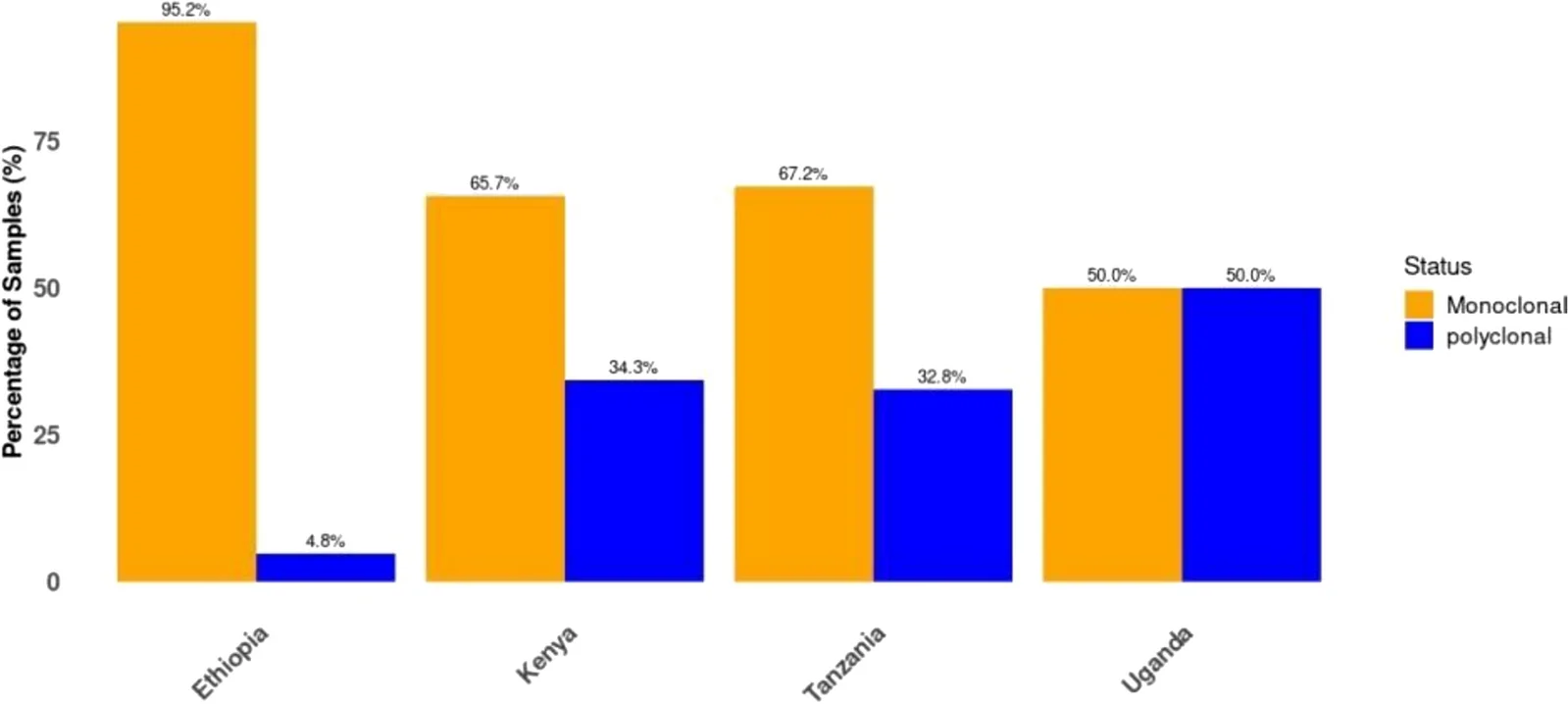
A bar plot showing infection clonality status across the study populations, used to assess within-host diversity based on the Fws metric. The x-axis represents the study populations, while the y-axis represents the percentage of sample. The orange color represents monoclonal infections while blue represents polyclonal infections

The inbreeding coefficient (*F*ws) was used to estimate the diversity of parasites present in an individual sample for every studied population. Isolates with the Fws value > 0.95 were considered to have monoclonal infections, while Fws < 0.95 indicated polyclonal infections. All the 1312 retained sequences were used for this analysis, whereby 875(66.7%) were reported to have clonal infection, while 437 (33.3%) had polyclonal infections. Most of the samples with monoclonal infections were from Kenya and Tanzania populations, where 453 (51.8%) samples came from Kenya (Kilifi = 424, Kisumu = 29) and 396 (45.3%) were from Tanzania (Kagera = 36, Kigoma = 87, Lindi = 43, Morogoro = 19, Tanga = 211). Of the samples with monoclonal infections, only 20 [(2.3%) (Amhara = 10 Oromia = 10) and 6 (0.68%, Apac)] samples were from Ethiopia and Uganda, respectively. The observed *Fws* values showed higher levels of clonal infections in Ethiopia (95.2%, 20/21) compared to other studied populations, including Kenya (65.7%, 453/690), Tanzania (67.2%, 396/589) and Uganda (50.0%, 6/12) (Fig. 2). For all the studied sub-populations, Amhara (100%) in Ethiopia reported more clonal infections followed by Oromia (90.9%), Tanga (71.0%), Kagera (69.2%), Kilifi (67.6%), Lindi (66.2%), Kigoma (60.8%), Morogoro (59.4%), Apac (50.0%), and Kisumu (46.0%) (Supplementary Figure S2). In the comparison of clonality assessment using *phistb*-based Fws and whole genome-based Fws generated by the MalariaGEN community, 66.7% of the 1312 samples were classified as monoclonal based on the *phistb*-derived Fws, whereas 54.7% were classified as monoclonal using the whole-genome approach (Supplementary Table S2). Statistical analysis showed a significant difference in the proportion of monoclonal samples between the *phistb*-based and whole-genome–based assessments (Supplementary Table S3). Overall agreement between the two methods was 81.7%, with most discrepancies arising from samples classified as polyclonal by the MalariaGEN whole-genome method but monoclonal by the *phistb*-based approach (Supplementary Table S4).

### Genetic diversity of the phistb gene

**Fig. 3:**
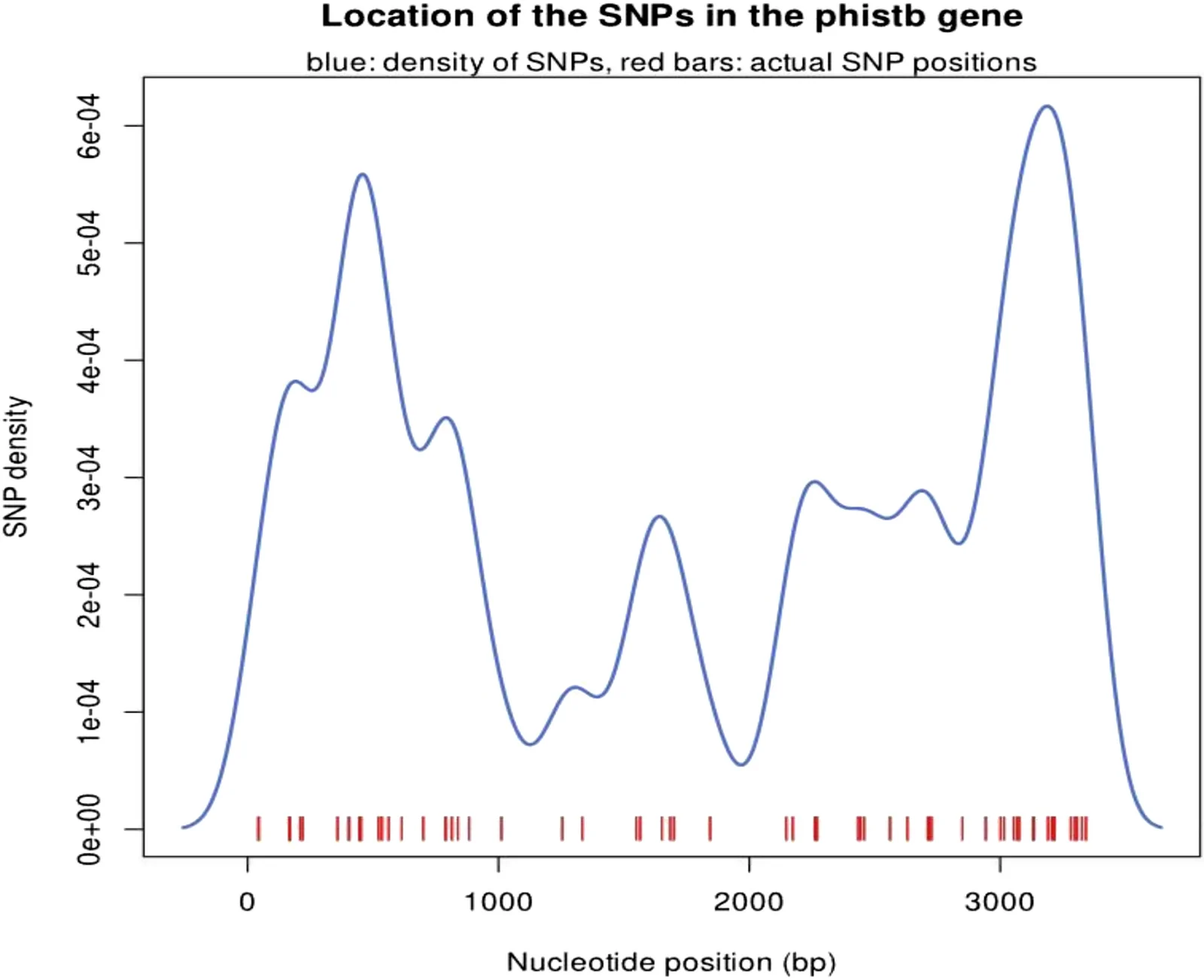
SNP density plot to indicates SNPs actual location along the phistb gene, the red bars indicate the actual position of SNPs while the blue line indicates the SNPs density

**Fig. 4:**
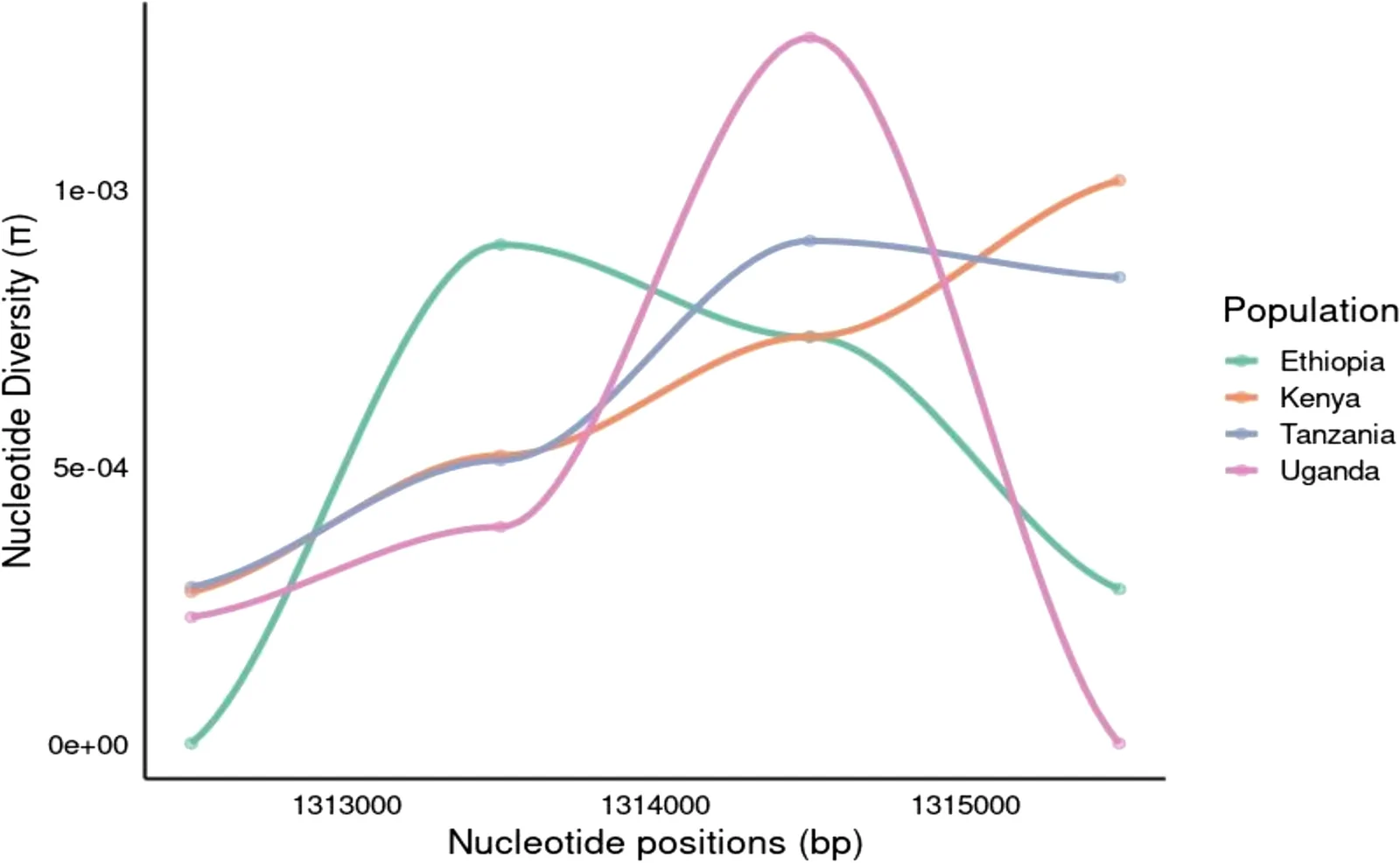
The plot of nucleotide diversity against nucleotide position for all the studied populations, each color corresponds to one studied population

To understand the level of genetic diversity within and between study populations, the *phistb* gene was analyzed across its full length, and summarized in a median joining haplotype network. SNPs density and distribution were assessed among all 1,312 sequences from Ethiopia (Amhara-10 and Oromoia-11), Kenya (Kilifi-627 and, Kisumu-63), Tanzania (Morogoro-32, Kagera-52, Kigoma-143, Lindi-65 and Tanga-297), and Uganda (Apac-12). The number of gene loci identified were 3364, of which 3360 (99.8%) did not have gaps, and so they were valid for genetic diversity analysis in the DnaSP. The transition to transversion mutation ratio was 0.818, for all the 1312 sequences combined together. For SNPs, 313 were identified along the *phistb* gene, and most of the SNPs appeared to have clustered around the 3000 base pair positions of the *phistb* gene (Fig. 3). Nucleotide diversity assessed in 500 bp sliding windows with 50 bp step size revealed relatively high values at the window midpoint position 1314000 in the Uganda population, compared to the other populations (Fig. 4). Different genetic diversity parameters were analyzed for each population and their subpopulations. The number of segregating sites was also determined for each studied population, and their corresponding sub-populations. The observed segregating sites were 9 in Ethiopia, 50 in Kenya, 38 in Tanzania and 7 in Uganda (Table 2). Correspondingly, the assessment of the number of segregating sites for each sub-populations showed a higher number of segregating sites in Kilifi (46) followed by Kigoma (29), Kisumu (18), Morogoro (16), Amhara (9), Apac (7), and Oromia (3) (Supplementary Table S5). Genetic diversity analysis showed slightly higher diversity in Kenya with the average number of nucleotide differences per DNA sequence (K) of 3.21 compared to other countries (Table 2).

**Table 2:** Genetic diversity metrics of the *phistb* gene among parasites from Ethiopia, Kenya, Tanzania and Uganda

Population	(n)	(s)	H	K	Π	Hd
Tanzania	589	38	98	3.14	0.095	0.87
Kenya	690	50	131	3.21	0.097	0.88
Uganda	12	7	6	2.39	0.072	0.87
Ethiopia	21	9	4	1.96	0.059	0.72

A summary of metrics assessed: *n* number of sequences, *s* number of segregating sites, *H* number of haplotypes, Average number of nucleotide differences(K), *π* nucleotide diversity, *Hd* haplotype diversity

**Fig. 5:**
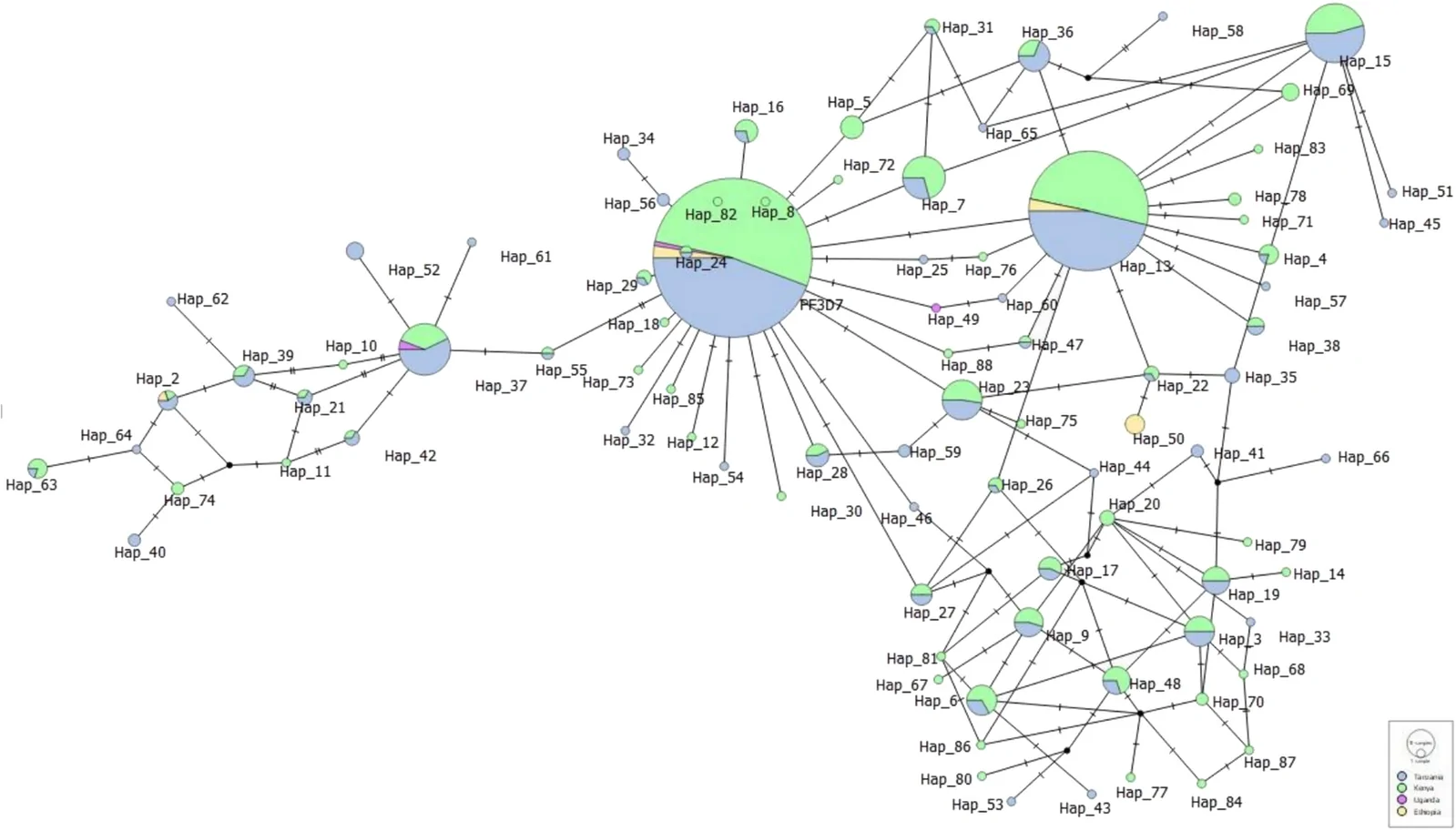
The median joining haplotype network provides an overview of the haplotypes obtained from 875 monoclonal sequences in Tanzania (396), Kenya (453), Uganda (6) and Ethiopia (20). Each circle represents the haplotypes, and the circles are colored to show the study populations. The size of the circle corresponds to the frequency of occurrence of each of the haplotype

A total of 88 haplotypes were identified in the 876 sequences analyzed which included 875 monoclonal samples and the PF3D7 reference sequence. Haplotype and nucleotide diversity among these sequences were 0.8 and 0.04, respectively. Up to 20 parsimony-informative sites were identified. These are the sites with at least two types of nucleotides, where at least one of the nucleotides occurs at a minimum frequency of two. Among the 88 haplotypes, haplotype 1 (hap_1) was renamed to PF3D7 as the reference gene belonged to this haplotype group. The PF3D7 haplotype, together with haplotype 13 (designed as Hap_13) were the most frequently observed haplotypes appearing in 332 (37.9%) and 188 (21.5%), samples, respectively, while the remaining haplotypes were unequally distributed in the rest of the study populations (Fig. 5).

### Population differentiation and structure of the *phistb* gene

**Fig. 6:**
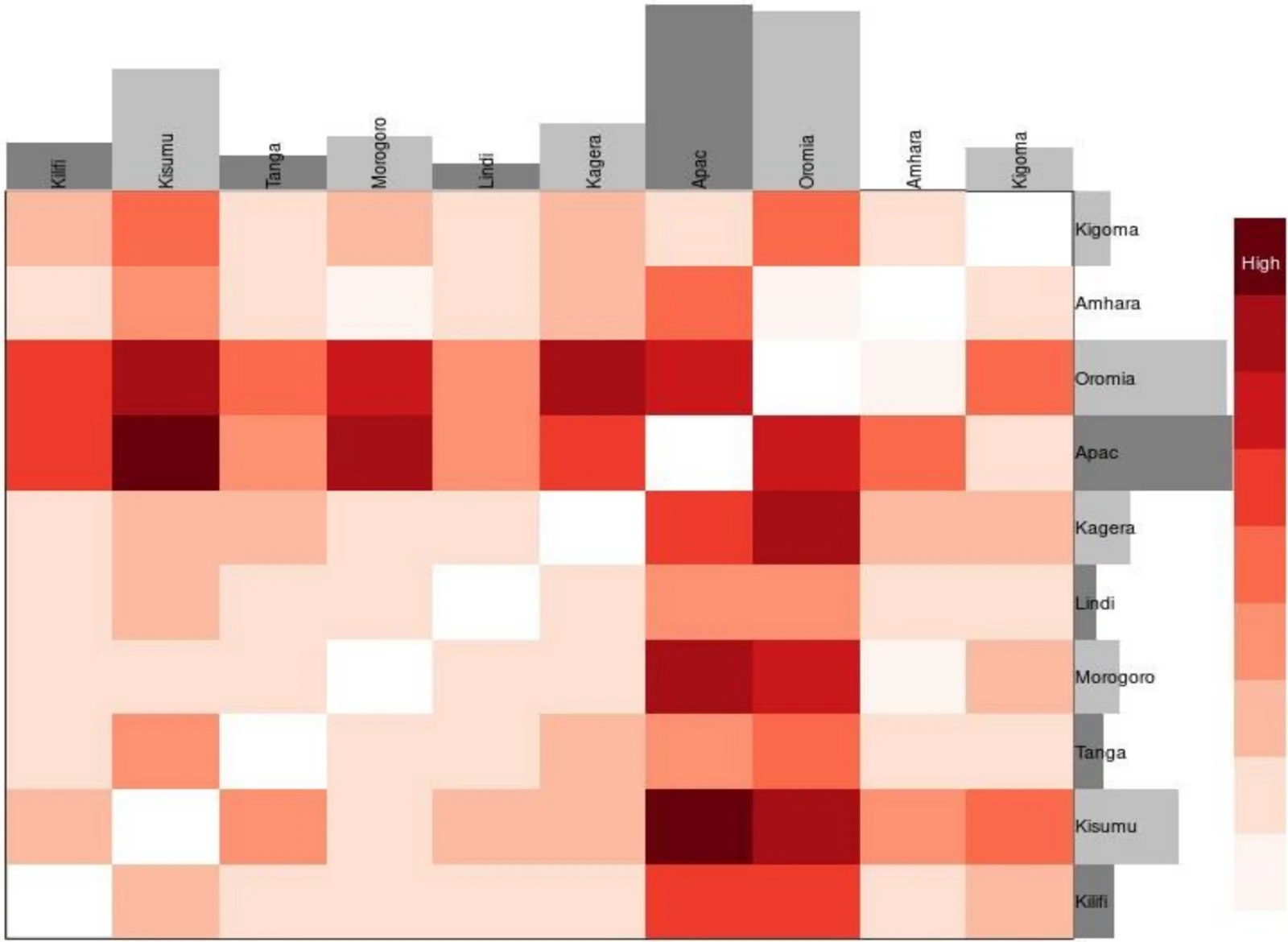
A heat map comparing genetic differentiation levels across the ten subpopulations studied. Fst values range from 0 to 1, the dark orange color indicating the highest genetic differentiation value observed between the compared subpopulations

To assess the level of genetic differentiation, the Fst statistic was assessed across all the study sub-populations using the pairwise Nei’s diversity function of the HierFstat package in R. The Fst values were categorised into four groups whereby, Fst values between 0 and 0.05 indicated low genetic differentiation, 0.05–0.15, moderate differentiation, 0.15–0.25, high differentiation and above 0.25 was very high genetic differentiation [18, 43, 58]. In this study, the Fst values were less than 0.05 in all the comparisons among the sub-populations, indicating low genetic differentiation across the sub-populations (Supplementary Table S5, Fig. 6). The population structure analysis did not show clear parasite clusters among the parasite populations, for all studied countries (Supplementary data, Figure S3), and for all the sub-populations (Supplementary data, Figure S4). The lack of clear separation of parasite population structure by PCA further confirms the observed low genetic differentiation among the parasite populations.

### Evidence of selection in the *phistb* gene

**Fig. 7:**
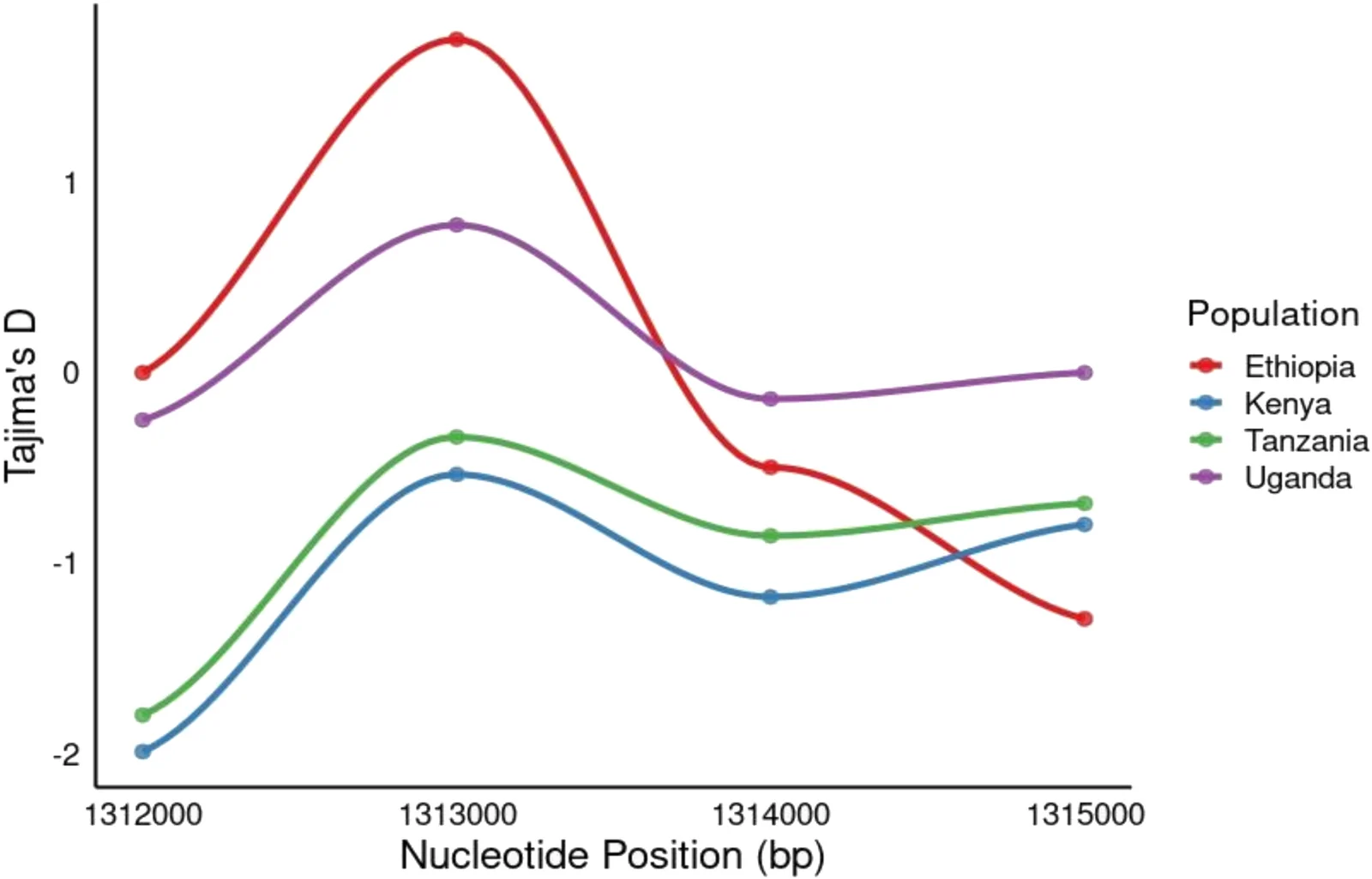
Tajima’s D plot in sliding windows of 1,000 bp, for all the 1312 sequences generated for the four populations from Tanzania, Kenya, Uganda and Ethiopia. The colored lines represent each of the study populations, with their corresponding Tajima’s D values

To assess whether selective forces are acting on the *phistb* gene, Tajima’s D and Fu and Li’s F and D tests, together with evaluation of the synonymous-to-nonsynonymous mutation ratio, were employed. These analyses helped determine the underlying selective forces and their impact on the *phistb* gene. Among the four countries (Ethiopia, Kenya, Tanzania, and Uganda), only the Ugandan population showed positive values for Tajima’s D and Fu & Li’s F and D tests (Table 3). Specifically, Tajima’s D values were −0.73 for Ethiopia, −1.46 for Kenya, 0.12 for Uganda, and −1.12 for Tanzania. Fu & Li’s F values were −1.58 for Ethiopia, −4.13 for Kenya, 0.35 for Uganda, and −2.35 for Tanzania, while Fu & Li’s D values were −1.76 for Ethiopia, −5.16 for Kenya, 0.35 for Uganda), and −2.62 for Tanzania (Table 3 and Fig. 7). Furthermore, Tajima’s D values at the synonymous and nonsynonymous sites were −1.136 and −1.545 respectively, resulting in a nonsynonymous-to-synonymous Tajima’s D ratio of 1.36 (Supplementary table S7).

**Table 3:** Neutrality tests values to detect evidence of selection among parasites from the four studied populations

Population	Tajima’s D	Fu & Li’ D	Fu & Li’s F
Tanzania	−1.12	−2.62	−2.35
Kenya	−1.46	−5.16	−4.13
Uganda	0.12	0.35	0.35
Ethiopia	−0.73	−1.76	−1.58

## Discussion

This study utilized freely available data from the MalariaGEN database and assessed the genetic diversity of the *phistb* gene in Tanzania and compared it with parasite populations from Ethiopia, Kenya, and Uganda. It also evaluated evidence of selection or neutrality using multiple neutrality tests (Tajima’s D, and Fu & Li’s F and D tests). Overall, the *phistb* gene exhibited low genetic diversity across all four countries, with no significant differences observed based on nucleotide diversity, haplotype diversity, and Wright’s fixation index (Fst). Low genetic differentiation was detected among populations, together with evidence of selection. The findings provide baseline information relevant for evaluating *phistb* as a potential malaria vaccine candidate in these countries that contribute substantially to the malaria burden in sub-Saharan Africa.

The Fws assessment showed higher levels of monoclonal infections in parasite populations from Ethiopia compared to the other studied populations. High levels of monoclonal infections are usually associated with low transmission intensities in a given region, and can also indicate low genetic diversity of parasites within the hosts [50]. Studies on malaria parasite prevalence and stratification in the East Africa, reports a spatial heterogeneity of malaria transmission with areas which are unsuitable for transmission [*P. falciparum* parasites prevalence rate (PfPR < 1%)] to areas of higher malaria transmission (PfPR > 30%) for both Tanzania, Kenya and Uganda [51]. This prior stratification study corresponds to the findings in this study, where Kenya and Tanzania had a comparable level of monoclonal infections (above 50%) signifying that the regions have heterogeneous transmission intensities with both areas of high and low transmission.

A relatively higher number of SNPs was observed along the 3000 base pair positions of the *phistb* gene compared to other regions of the gene. This pattern could suggest selection pressure targeting the gene loci [19]. Similar trends of high levels of polymorphisms at specific gene loci were reported in previous studies that assessed the genome-wide variations and identification of vaccine targets in the *P. falciparum* genome. In the previous studies, genomic regions with peaks of polymorphism, and/or accumulation of many polymorphic genes were thought to be antigenic, and also the regions were suspected as drug targets, thus they can be used in malaria vaccines development [52, 53].

Parasites from Kenya showed slightly higher nucleotide differences per sequence (K) and nucleotide diversity (π) compared to the other countries. This contrasts with previous reports describing generally low diversity of *phistb* across African populations [25]. Differences between studies may partly reflect variation in sample size as previously reported [54, 55]. Sliding window analysis further identified specific regions within the gene exhibiting relatively higher diversity (Fig. 4), suggesting localized allelic variation that may be relevant when considering antigenic diversity [15, 19, 50].

Among the 875 monoclonal samples analyzed, 88 haplotypes were identified. Two haplotypes (Hap_1/PF3D7 and Haplotype_13) accounted for 59% of all observed haplotypes across the four countries. Although Kenya showed the highest number of haplotypes, two predominant haplotypes were consistently shared across populations, consistent with previous reports of limited dominant *phistb* haplotypes in sub-Saharan Africa [25]. Despite many haplotypes that were observed individually in different study populations, the level of genetic diversity remained low across all the study populations, as attested by the population structure analysis and the genetic differentiations. The limited variability of the *phistb* haplotypes further supports its suitability for its inclusion in malaria vaccine development.

Low genetic differentiation, defined as Fst < 0.05, was observed in the Fst comparisons across all the sub-populations (Supplementary Table S6, Fig. 6), despite the spatial heterogeneity of malaria transmission among the studied populations, and the geographical distance among populations. Similarly, principal component analysis did not reveal a clear population structure (Supplementary Figure S3 & 4 respectively). Similar findings of low differentiation among malaria parasites populations despite heterogeneous transmission intensities and geographical isolation have been reported in other African settings. The lack of genetic differentiation, and population structure could indicate a high genetic exchange or gene flow among these populations as a result of human and vector migration within and among these countries [56–59].

The Tajima’s D test values together with the Fu & Li’s F and D tests reported negative values for all malaria parasites populations except the Uganda parasites, indicating an excess of rare alleles or rare mutations within parasites from Ethiopia, Kenya and Tanzania. In Uganda, positive Tajima’s D, Fu and Li’s F & D test values were observed indicating an excess of intermediate frequency alleles that is usually associated with balancing selection [60–62]. These patterns differ from previous findings [25], and may partly reflect differences in sampling depth and population representation. Previous studies reported that small sample sizes affect the Tajima’s D and Fu & Li’s F and D tests because the most common neutrality tests (Tajima’s D) is a product of the number of segregating sites (S) and nucleotide diversity, thus the use of large number of sequences helps to capture all the differences among species resulting in more reliable tests results [54, 55]. Moreover, the departure from neutrality in the nonsynonymous sites (Tajima’s D = −1.545) was stronger than in the synonymous sites (Tajima’s D = −1.136), (Supplementary Table S7). This suggests that nonsynonymous mutations are more skewed towards rare variants than the synonymous ones, consistent with recent population expansion or purifying selection [49].

This study had relatively small numbers of samples from Ethiopia and Uganda, which may limit the genetic diversity observations made at that time point. However, all the available data set from the MalariaGEN database (https:www.malariagen.net/) was effectively used to generate all the necessary information and the findings were cross-checked through a repeated analysis between the R software and the DnaSP software, and the obtained findings were consistent in both the analysis tools.

## Conclusion

This study reports low genetic diversity of the *phistb* gene in Tanzania and its neighboring countries of Kenya, Uganda and Ethiopia, despite varying malaria transmission intensity among them, thus making it a suitable candidate gene for malaria vaccine. Further studies should be conducted to assess individual antibodies recognition of the *phistb* variants and the ability to elicit cross reactivity to further support its potential as a vaccine candidate. Additionally, given that the *phistb* gene is highly conserved among the main human malaria parasites, PHISTb proteins could be explored as alternative diagnostic markers to ensure more reliable test results, particularly, in regions where mutations in traditional markers like *hrp2* and *hrp3* genes compromises diagnostics accuracy.

## Supplementary Information

Supplementary material 1.

## Data Availability

This study analyzed data from the MalariaGEN *Plasmodium falciparum* community project (Pf7). The data is publicly available under the MalariaGEN terms of use for the *P. falciparum* community project: https://www.malariagen.net/data_package/open-dataset-plasmodium-falciparum-v70/. The analysis codes are available at: https://github.com/ruth260/Genetic-Diversity-Analysis-of-the-PHISTb-gene-in-Plasmodium-falciparum.

## Abbreviations

AMA1: Apical membrane antigen 1
DDH: District designated hospital
DNA: Deoxyribonucleic acid
*Pfemp1*: *P.falciparum* Erythrocyte membrane protein1
*glurp*: Glutamate rich protein
G6PD: Glucose 6 phosphate dehydrogenase
GPI: Glycophosphatidylinositol
*hrp2/3*: Histidine-rich protein 2/3
IPTp: Intermittent preventive treatment in pregnancy
IPTsc: Intermittent preventive treatment in school children
LLIN: Long-lasting insecticide treated nets
LSA 1: Liver-stage antigen 1
LSM: Larval source management
LYM: Lysine-rich membrane associated *phistb* gene
MSMT: Molecular surveillance of malaria in Tanzania
*msp1*: Merozoite surface protein1
NMCP: National malaria control program
NIMR: National Institute for Medical Research
PCA: Principal component analysis
PDNA: Pathogen diversity network Africa
*phist*: *Plasmodium* Helical interspersed subtelomeric gene
PRESAN: *Plasmodium* Ring-infected erythrocyte surface antigen N-terminal
RBC: Red blood cell
RDT: Rapid diagnostic test
RTS, S: Repeat T-cells Surface antigen
SNP: Single nucleotide polymorphism
SSA: Sub-Saharan Africa
TES: Therapeutic efficacy study
TRAP: Thrombospondin-related protein.
VCF: Variant calling format file
WGS: Whole genome sequencing
WHO: World health organization
