# Supplementary material for: Genetic diversity of Plasmodium falciparum helical interspersed subtelomeric (phistb) gene in Tanzania and neighboring countries
Source: Malar J. 2026 Jun 24;25:327. doi: 10.1186/s12936-026-06011-x (PMC13555929; doi:10.1186/s12936-026-06011-x)
Supplement: Supplementary file 1 [file 12936_2026_6011_MOESM1_ESM.docx]

**Table S1. Details on previous studies from which the analyzed samples were obtained**

| **Country** | **Study site** | **Study ID** | **Year** | **Study design** | **Study participants** |
| --- | --- | --- | --- | --- | --- |
| Tanzania | Muheza designated district hospital, and Morogoro Regional Hospital | 1007-PF-TZ-DUFFY | 2010 | Longitudinal, and Cross-sectional study designs | Pregnant women, children and infants |
|  | Muleba, Muheza and Nachingwea | 1095-PF-TZ-ISHENGOMA | 2013 | Clinical trial (Therapeutic Efficacy Studies) | Patients attending clinics/hospitals |
|  | Muheza Designated District hospital and Ujiji Health Centre | 1167-PF-TZ-ISHENGOMA-SM | 2014 | Randomized clinical trial | Infants (6 months) and children aged up to 10 years with uncomplicated Malaria |
| Kenya | Kilifi | 1015-PF-KE-NZILA | 2007 | Cross-sectional studies | Children with acute febrile malaria |
|  | Kilifi | 1027-PF-KE-BULL | 2007 | Not Available | Not Available |
|  | Kisumu | 1107-PF-KEN-KAMAU | 2014 | Cross-sectional studies | Patients with uncomplicated malaria aged between 6 months and 65 years |
|  | Kilifi | 1132-PF-K1000G-DBS-KE-BEJON | 1994 - 2018 | Longitudinal cohort studies | Symptomatic and asymptomatic participants in Kilifi regions |
| Uganda | Apac | 1024-PF-UG-BOUSEMA | 2010 | Cross-sectional and longitudinal studies | Children and adults residing in malaria endemic region |
| Ethiopia | Amhara, Oromia | 1098-PF-ET-GOLASSA | 2013-2015 | Cross-sectional studies | *P. falciparum* malaria patients |

# Assessment of per-site missingness across the *phistb* gene

The biallelic filtered VCF data was used to assess per-site missingness of the *phistb* gene. VCFtools was used to assess the per-site missingness of the *phistb* gene and the observed values were plotted as a fraction of per-site missingness against genomic positions (**Supplementary figure 1**).

**Figure S1.** Fraction of missing data per site across genomic positions along the phistb gene. Each dot represents an individual genomic position, plotted by its location within the gene (x-axis) and the fraction of SNPs with missing genotype data at that site.

# Comparison of clonality estimations between MalariaGEN whole genome-based,and phistb-based estimations

MalariaGEN provides a whole genome-based estimation of clonality. To have a deeper understanding of the clonality, we analyzed clonality based on *phistb* gene using the FWS metrics. Our *phistb*-based values were then plotted for each subpopulation (**supplementary figure2**), and compared to those generated by the MalariaGEN. The proportion of samples classified as monoclonal differed significantly between the two approaches (54.7% for MalariaGEN and 66.7% for *phistb*-based estimates; two-sample test for equality of proportions, p < 0.001), indicating a systematic difference in classification. Agreement between methods was assessed using a 2 × 2 contingency table comparing sample-level classifications. Overall agreement was 81.63%, with most discrepancies arising from samples classified as polyclonal by MalariaGEN but monoclonal by the *phistb*-based approach (**Supplementary Table 1, 2 &3**), likely due to methodological differences (whole-genome vs gene-based estimation).

**Table S2. Clonality Classification Using MalariaGEN Whole-Genome Fws and *phistb*-Based Fws**

|  | **MalariaGEN_Fws (n, %)** | **phistb-based_Fws (n, %)** |
| --- | --- | --- |
| **Monoclonal** | 718(54.73%) | 875 (66.7%) |
| **Polyclonal** | 594(45.27%) | 437 (33.3%) |
| **Total** | 1312 | 1312 |

**Table S3. Comparison of Clonality assessment using MalariaGEN Whole-Genome Fws and *phistb*-Based Fws**

| **Comparison** | **Proportion (Monoclonal)** | **x-squared** | **Df** | **95%CI (difference)** | **p-value** |
| --- | --- | --- | --- | --- | --- |
| MalariaGEN vs phistb-based clonality | 0.547 vs 0.667 | 38.88 | 1 | −0.158 to −0.082 | 4.50 × 10⁻¹⁰ |

**Table S4. Agreement between MalariaGEN and *phistb*-based methods for clonality assessment.**

| ***phistb*-based estimation** | **MalariaGEN-based estimation** | |
| --- | --- | --- |
|  | **Monoclonal** | **Polyclonal** |
| **Monoclonal** | 676 | 42 |
| **Polyclonal** | 199 | 395 |

Agreement: (Number of agreements)/Total observation

((676 + 395)/1312) * 100 = 81.63%

**Figure S2.** A bar plot showing infection clonality status across the study sub-populations, used to assess within-host diversity based on the FWS metric. The x-axis represents the study sub-populations, while the y-axis represents the percentage of sample. The orange color represents Monoclonal infections while blue represents a polyclonal infection.

**Table S5.** **Genetic diversity metrics of the *phistb* gene among the Ethiopia, Kenya, Tanzania, and Uganda sub-populations.**

| Population | (n) | (s) | H | K | π | Hd |
| --- | --- | --- | --- | --- | --- | --- |
| Amhara | 10 | 9 | 4 | 2.73 | 0.000 | 0.77 |
| Apac | 12 | 7 | 6 | 2.39 | 0.000 | 0.87 |
| Kagera | 52 | 14 | 20 | 3.49 | 0.001 | 0.88 |
| Kigoma | 143 | 29 | 67 | 3.26 | 0.000 | 0.87 |
| Kilifi | 627 | 46 | 115 | 3.17 | 0.009 | 0.88 |
| Kisumu | 63 | 18 | 30 | 3.65 | 0.001 | 0.92 |
| Lindi | 65 | 15 | 23 | 2.81 | 0.000 | 0.84 |
| Morogoro | 32 | 16 | 16 | 3.8 | 0.001 | 0.9 |
| Oromia | 11 | 3 | 3 | 1.38 | 0.000 | 0.72 |
| Tanga | 297 | 23 | 60 | 3.15 | 0.009 | 0.87 |

**Table S6. FST values for genetic differentiation among the ten subpopulations studied**

|  | **Kilifi** | **Kisumu** | **Tanga** | **Morogoro** | **Lindi** | **Kagera** | **Apac** | **Oromia** | **Amhara** | **Kigoma** |
| --- | --- | --- | --- | --- | --- | --- | --- | --- | --- | --- |
| **Kilifi** | 0 | 0.0016 | 3.00E-04 | 0 | -6.00E-04 | -5.00E-04 | 0.0054 | 0.0059 | -1.00E-04 | 0.0011 |
| **Kisumu** | 0.0016 | 0 | 0.0021 | -1.00E-04 | 0.0017 | 4.00E-04 | 0.0121 | 0.0097 | 0.0023 | 0.0044 |
| **Tanga** | 3.00E-04 | 0.0021 | 0 | 2.00E-04 | -5.00E-04 | 4.00E-04 | 0.0028 | 0.0051 | -9.00E-04 | 2.00E-04 |
| **Morogoro** | 0 | -1.00E-04 | 2.00E-04 | 0 | 3.00E-04 | -0.0012 | 0.0091 | 0.0072 | -0.0023 | 0.0019 |
| **Lindi** | -6.00E-04 | 0.0017 | -5.00E-04 | 3.00E-04 | 0 | -2.00E-04 | 0.0037 | 0.0037 | -0.001 | 2.00E-04 |
| **Kagera** | -5.00E-04 | 4.00E-04 | 4.00E-04 | -0.0012 | -2.00E-04 | 0 | 0.0068 | 0.0099 | 0.0014 | 0.0015 |
| **Apac** | 0.0054 | 0.0121 | 0.0028 | 0.0091 | 0.0037 | 0.0068 | 0 | 0.008 | 0.0045 | 0 |
| **Oromia** | 0.0059 | 0.0097 | 0.0051 | 0.0072 | 0.0037 | 0.0099 | 0.008 | 0 | -0.003 | 0.0039 |
| **Amhara** | -1.00E-04 | 0.0023 | -9.00E-04 | -0.0023 | -0.001 | 0.0014 | 0.0045 | -0.003 | 0 | -0.0012 |
| **Kigoma** | 0.0011 | 0.0044 | 2.00E-04 | 0.0019 | 2.00E-04 | 0.0015 | 0 | 0.0039 | -0.0012 | 0 |

***Figure S3****. A plot on the first and second Principal components (PC1 & PC2) for Tanzania, Kenya, Uganda, and Ethiopia parasites population. The shape and color of the dots represents the population and country where the samples were taken.*

***Figure. S4****. Plot of the first and second Principal components (PC1 & PC2) for parasites collected from all sub-populations in the four countries, each color used indicates one subpopulation.*
